# A Review of Weathering Studies in Plastics and Biocomposites—Effects on Mechanical Properties and Emissions of Volatile Organic Compounds (VOCs)

**DOI:** 10.3390/polym16081103

**Published:** 2024-04-16

**Authors:** Monwabisi Cyril Nzimande, Asanda Mtibe, Shepherd Tichapondwa, Maya Jacob John

**Affiliations:** 1 Centre for Nanostructures and Advanced Materials, Chemicals Cluster, CSIR, Pretoria 6011, South Africa; monwabisinzimande6@gmail.com (M.C.N.); amtibe@csir.co.za (A.M.); 2 Water Utilization and Environmental Engineering Division, Department of Chemical Engineering, University of Pretoria, Pretoria 0028, South Africa; shepherd.tichapondwa@up.ac.za; 3 Department of Chemistry, Nelson Mandela University, Port Elizabeth 6031, South Africa

**Keywords:** weathering, aging, carbonyl index, plastics, biopolymers, biocomposites, VOCs

## Abstract

Polymeric materials undergo degradation when exposed to outdoor conditions due to the synergistic effects of sunlight, air, heat, and moisture. The degradation can lead to a decline in mechanical properties, fading, surface cracking, and haziness, attributed to the cleavage of the polymer chains and oxidation reactions. Accelerated weathering testing is a useful technique to evaluate the comparative photodegradation of materials within a reasonable timeframe. This review gives an overview of the different degradation mechanisms occurring in conventional plastics and bio-based materials. Case studies on accelerated weathering and its effect on the mechanical properties of conventional plastics and biocomposites are discussed. Different techniques for analysing volatile organic emissions (VOCs) have been summarized and studies highlighting the characterization of VOCs from aged plastics and biocomposites after aging have been cited.

## 1. Introduction

The transition to circular economies in the plastic sector offers promising solutions to combat plastic pollution, reduce carbon emissions, and promote a more sustainable future. This includes increasing recycling rates, designing products for circularity and developing environmentally friendly alternatives. As part of increasing recycling rates, there has been a rise in the development of recycled products for outdoor applications, such as plastic timber products [outdoor furniture (benches in school playgrounds), decking] and the use of plastic waste in asphalt to improve the durability of roads. This is considered as a promising strategy, as it converts waste plastics into value-added durable products and large quantities of waste plastics are diverted from landfills. It is known that these products are exposed to dynamic outdoor conditions (the influence of sunlight, temperature and humidity) for long durations, leading to photo-degradation, which can result in surface changes (loss of colour, cracking) and a loss of mechanical properties. Apart from the decline in mechanical properties, the degradation of polymers results in the generation of volatile organic compounds (VOCs), which are toxic and can have adverse short-term and long-term effects on human health. Several studies have dealt with the analysis of mechanical and physical properties of plastics when subjected to degradation processes; however, reports on emissions of VOCs from the degradation of plastic products are limited.

The other strategy to tackle plastic pollution is the use of bio-based materials for niche products. A major portion of the generated plastic wastes consists of single-use products, which are not recycled due to economic reasons or contamination with soil (e.g., mulch), food waste (e.g., food cartons) and biomedical waste (e.g., syringes, surgical aprons, diagnostic kits), and can be replaced using bio-based materials (biopolymers and biocomposites). While biopolymers possess environmental advantages over conventional plastics in terms of the sustainability of raw materials and the embodied energy in production processes, there are limited studies on the environmental impact of degradation products that are formed when biopolymers are subjected to weathering. The tracking of degradation products is necessary towards ensuring the sustainability of biopolymers and making sure that toxic chemicals/additives/plasticizers, if used in the production stages, are not generated as byproducts.

It is clear that there is a critical gap in the understanding of the long-term environmental impact of conventional plastics and biopolymers; most of the degradation studies on plastics deal with the effect of thermal or environmental aging on the physical and mechanical properties of plastics [1]. Studies on the analysis of emissions of volatile organic compounds (VOCs) during degradation in plastics and biopolymers are limited and need to be addressed. This review provides a detailed account of studies on accelerated weathering and its effect on the mechanical properties in conventional plastics and bio-based materials. Additionally, the different techniques of analysing volatile organic emissions (VOCs) and studies on characterizing VOCs from aged plastics and biocomposites are also highlighted.

## 2. Plastics Degradation

Polymer degradation refers to changes in polymer properties due to chemical, physical, or biological reactions that result in bond scissions and subsequent chemical transformations [2]. Degradation leads to changes in the mechanical, thermal, and optical properties of the material, such as crazing, cracking, erosion, discoloration, and phase separation. Depending on the causative agents, polymer degradation can be classified primarily into three categories: photo-oxidative degradation, thermal degradation, and biodegradation.

### 2.1. Thermal Degradation

Thermal degradation involves degradation occurring in plastics when exposed to high temperatures for long durations. It involves a series of thermo-oxidative reactions resulting in the breaking of long polymer chains and the release of radicals [3]. The parameters affected are molecular weight, molar weight distribution, degree of crystallinity, and degree of branching.

### 2.2. Photodegradation

Photodegradation is degradation occurring in materials when exposed to sunlight. Sunlight comprises ultraviolet, visible, and infrared radiation, of which UV radiation is responsible for degradation in polymers. UV radiation has three components: UVA, which has a wavelength range of 320–400 nm, UVB, with a range of 280–295 nm, and UVC, with a range of 100–280 nm [4]. The UV-B is the primary radiation that initiates the photooxidative degradation of common polymers such as LDPE [5].

The visible and infrared component in sunlight can cause colour changes, fading, and increased temperatures in plastics [6]. Photodegradation results in a decrease in the performance of materials, altering their physical and optical characteristics, including changes in molecular weight, the deterioration of mechanical properties, loss of colour and surface finish, as well as changes in mechanical properties [7]. After end of life, plastic products will be exposed to a combination of two or more factors (e.g., temperature and humidity), often resulting in complex synergistic degradation of the material.

Amongst the different types of polymers, the most stable are the polyfluorocarbons [poly (vinylidene difluoride) (PVDF), poly (tetrafluoroethylene (PTFE)], silicones, and polymethylmethacrylate (PMMA) [8]. These polymers do not absorb UV light and are difficult to oxidize. In the case of thermoplastic polyolefins (polyethylene and polypropylenes), photodegradation proceeds via oxidation, leading to the destruction of their properties. Upon weathering, thermoplastic polyolefins do not undergo yellowing; however, the surface becomes cracked after prolonged exposure [9]. Polyvinylchlorides (PVC) do not absorb UV light; however, they undergo rearrangement by losing an HCl molecule and forming double bonds, resulting in yellowing and brittleness [10]. It is highly recommended to use catalysts such as hindered amine light stabilizers (HALS), which can terminate the reactions. The addition of UV-absorbing colouring pigments such as carbon black, titanium dioxide, and iron oxides, also provides protection [11]. The use of these pigments in high concentration restricts the UV penetration into the surface, resulting in less colour loss and a lower rate of degradation [12].

In the case of aromatic polymers, such as polystyrenes, polyesters, and polycarbonates, absorption of UV light leads to the oxidation of the aromatic rings and the formation of coloured products. The addition of UV absorbers and HALS is not seen to be as effective as in aliphatic polymers [13].

Even in polymers (e.g., polyethylene) that do not absorb UV, the presence of impurities or chromophores can trigger photo-oxidative degradation. UV stabilizers, such as HALS, are commonly added to polymers to protect them from sunlight-induced degradation, extending their lifetime in exposed applications [14]. The stability performance during photo-oxidation of a formulation depends on the individual contribution of each stabilizer, as well as the respective interactions between components. The mechanism of action of photo-stabilisers usually involves the following: prevention of light absorption by polymer-bonded chromophores and impurities, deactivation of excited states of chromophores through fast quenching, and trapping of formed free radicals through combination reactions [15]. The common types of photo-stabilizers used in polymers are presented below.

#### 2.2.1. UV Screeners

Typical examples include pigments, such as carbon black, ZnO, TiO_2_, MgO, CaCO_3_, and BaSO_4_. In the case of carbon black, the average size of the primary particles of carbon black determines its UV absorption capability. Carbon black with smaller primary particles have a larger surface area for optical absorption, resulting in higher UV absorbance. In a study conducted by Ammala et al., the authors observed that the inclusion of 4% TiO_2_ significantly slowed down the deterioration of PP under UV exposure from 85% to 46% [16].

#### 2.2.2. UV Absorbers

Typical stabilizers acting as UV absorbers are phenyl salicylates, 2-hydroxybenzo-phenones, coumarin derivatives, and resorcinol esters [17]. Rabie et al. [18] carried out a study to investigate how enhancing the photostability of polystyrene (PS) against UV-induced degradation might be attained. To investigate possible synergistic effects resulting from their various modes of action in the photo stabilization process, these substances were also mixed with commercial UV absorbers, including phenyl salicylate and a derivative of 2-hydroxybenzophenone. The study also evaluated the level of discoloration. The results revealed that the studied materials had a higher stabilizing effectiveness than commercial UV absorbers such as phenyl salicylate and 2-hydroxybenzophenone.

#### 2.2.3. Excited State Quenchers

Quenchers, which include nitrobenzene and halogens, are often used to deactivate long-lived excited states of chemicals such as ketones and aldehydes. This deactivation process causes the excited state’s energy to be released as heat [19].

#### 2.2.4. Free Radical Scavengers and/or Hydroperoxide Decomposers

Sulphur-containing metal complexes such as dialkyldithiocarbamates, dialkyldithiophosphates, and thiobisphenolates, are highly effective in decomposing hydroperoxide groups, even when used in catalytic proportions. These metal complexes have been shown to be particularly effective UV stabilizers for polypropylene (PP) and can lower the hydroperoxide content of peroxidised PP films even at room temperature [20].

### 2.3. Biodegradation

The term biodegradation refers to degradation by microorganisms such as bacteria and fungi into water, naturally occurring gases such as carbon dioxide (CO_2_), water and new microbial biomass production. The biodegradation of polymeric materials mainly depends on various environmental factors, such as active microbial species present in environments, temperature, humidity, the presence of oxygen, pH level, and UV radiation [21]. The physical and chemical properties, such as polymer surface area, hydrophobic and hydrophilic nature (which can be determined by inexpensive techniques) [22,23], chemical nature, molecular weight, molecular weight distribution, crystallinity, crystal structure, glass transition temperature, melting temperature, and elasticity of materials, also play important roles in the polymer biodegradation process. The rate of biodegradation of a biodegradable plastic may be different in soil, in humid or dry climate, in surface water, in marine water, or man-made organic recycling systems such as home composting, industrial composting, or anaerobic digestion [24]. For instance, biodegradable plastic degrades faster in industrial composting in the presence of oxygen, microorganisms, and high temperatures (50–60 °C). According to the EN13432 standard [25], in industrial composting conditions, to be claimed as biodegradable, 90% of carbon must be converted to CO_2_ in 6 months, whereas in home composting conditions, biodegradation should reach at least 90% in 12 months.

Apart from mechanical deterioration, it is well established that the degradation of polymers results in the generation of volatile organic compounds (VOCs), such as lactones, esters, ketones, and carboxylic acids, because of the long-term degradation of polyolefins and subsequent reduction in molecular weight [26]. These released VOCs can undergo complex photochemical reactions, leading to the formation of oxidized compounds. Furthermore, VOCs are generally toxic and can have detrimental short-term and long-term effects on human health. Only a limited number of studies have specifically focused on analysing the VOCs emitted from plastics during degradation. For instance, recent research [27] showed that plastic wastes can generate VOCs of approximately 500 μg/g at 200 °C. Another recent study found that environmentally aged low-density polyethylene (LDPE) released various types of hydrocarbon gases, including methane, with the production rate being dependent on the surface-to-volume ratio [28]. This indicates that the release of VOCs can continue indefinitely throughout the lifespan of plastics, as long as they remain exposed to various degradation conditions.

Many studies on plastic degradation have focused on the impact of thermal or environmental aging on the physical and mechanical properties of plastics, with limited quantitative data available on the release of VOCs during degradation. The potential release of VOCs during plastic degradation is concerning due to their harmful effects on human health and their contribution to air pollution. Therefore, a better understanding of the mechanisms and factors that influence VOC emissions during plastics degradation is crucial. The following sections aim to address this knowledge gap by providing a recent account of studies on the effect of environmental aging on the mechanical properties of materials (plastics and biocomposites) and the analysis of VOCs emissions during plastics degradation.

## 3. Natural vs. Accelerated Weathering

There are two main types of weathering studies: studies on natural weathering and accelerated weathering [29].

### 3.1. Natural Weathering

Natural weathering studies deal with the practice of exposing polymer samples to outdoor environmental conditions over an extended period, typically ranging from months to years. The samples are placed in an outdoor environment and monitored for changes in properties, such as mechanical strength, colour, surface texture, and chemical composition [30]. Studies on the natural weathering of sugarcane leaf-reinforced epoxy composites have been reported. These studies involved outdoor exposure for 3 months, resulting in a subsequent decrease in tensile properties [31].

### 3.2. Accelerated Weathering

Accelerated weathering studies use artificial methods to simulate the effects of outdoor weathering on polymers in a shorter period. Accelerated weathering chambers typically expose polymer samples to controlled environmental conditions, such as UV radiation, heat, moisture, and other factors, in a laboratory setting [32]. These tests aim to accelerate the degradation processes that occur in natural weathering and provide quicker results, compared to natural weathering studies. Accelerated weathering studies allow researchers to study the effects of specific environmental factors on polymer degradation and assess the performance of polymers under accelerated ageing conditions. The common testing standards include ASTM G155 (877, 2009, ASTM, 2005) [33], which reproduces the weathering effects occurring when materials are exposed to sunlight (either direct or through window glass) and moisture. Additionally, ASTM D7869 (International, 2017) [34] offers the Xenon Arc Exposure Test with Enhanced Light and Water Exposure for Transportation Coatings, including conditions such as irradiance of 0.75 W/m^2^/nm at 340 nm, with the duration of testing ranging from 500 to 1000 h.

The photooxidative degradation of acrylonitrile-butadiene-styrene (ABS) polymer using both outdoor exposure and accelerated ageing methods was recently investigated [35] to study the correlation factor between these two ageing strategies. FTIR analysis and mechanical property tests were used to evaluate the impacts of photo-oxidation. The mechanical performance of ABS samples decreases as the amount of carbonyl increases for samples exposed to outdoor weathering and accelerated weathering; however, the formation of cracks on the surface of ABS samples was more pronounced in the samples exposed to outdoor weathering. The results, which demonstrated strong agreement, led to the conclusion that 1260 h of exposure under a filtered xenon lamp (with a wavelength range of 300–400 nm) at 48 °C and 50% relative humidity successfully replicated the effects of one year of outdoor exposure in Lisbon.

## 4. Case Studies on Weathering

### 4.1. Plastics

The main effects of weathering are the breaking of polymer chains, leading to a reduction in molecular weight and mechanical properties. In certain polymers, such as polyethylene, crosslinking may occur, leading to decreased ductility. Other effects on prolonged weathering include surface erosion in the form of cracking and chalking, yellowing, and fading of colour [13]. In polyolefins, photo-degradation occurs via the introduction of chromophores, such as hydroperoxide groups, carbonyl groups, and double bonds. Among these, carbonyl groups are the main agents responsible for the photochemical-induced degradation. The further degradation of the carbonyl groups proceeds by the Norrish I reaction, leading to termination via either crosslinking or chain scission. Alternatively, degradation can also advance by the Norrish II reaction, whereby carbonyl groups and terminal vinyl groups are produced, and chain scission occurs. Hence, there are two mechanisms—chain scission and crosslinking—occurring during the course of accelerated polyethylene photodegradation.

In the studies elaborated below, the changes after weathering are usually confirmed as increased intensity of carbonyls groups in a Fourier Transform Infrared (FTIR) spectrum. The carbonyl index (CI) is defined as the ratio of the peak intensity of the carbonyl groups, at 1750 cm^−1^, to the peak of the methyl group, at 3000–2800 cm^−1^ or 1450 cm^−1^. The CI provides a measure of degradation because carbonyl, vinyl, and hydroperoxides are the prominent products formed by photodegradation, which increase with ageing. These products are found in the carbonyl region of the FTIR spectra.

**Figure 1:**
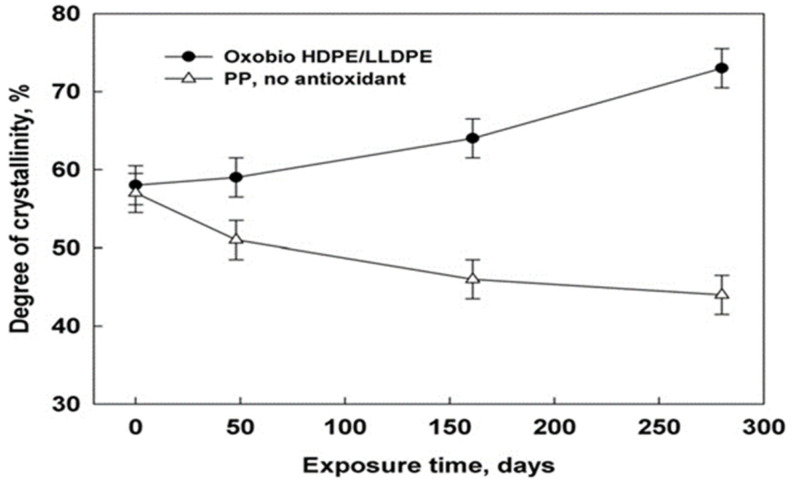
Degree of crystallinity of PP samples without antioxidant compared to a commercial oxo-biodegradable HDPE/LLDPE sample as a function of exposure time.

In a study reported by Ojeda et al. [36], the abiotic degradability of extruded blown films made from HDPE, LLDPE, PP with low or zero antioxidant additive concentrations, and an HDPE/LLDPE blend with a pro-oxidant additive (oxo-bio HDPE/LLDPE blend) were investigated. The samples were exposed to natural weathering for a year and the thermal and mechanical properties were studied. Figure 1 shows the decreasing crystallinity of the PP samples (without antioxidant) during exposure, whereas the crystallinity of the oxo-bio-HDPE/LLDPE blend was found to increase. These opposing crystallinity patterns were attributed to the decreasing molecular size in the blend and greater amount of impurities in PP. The oxo-biodegradable blend exhibited reduced mechanical properties at a slower rate than both PP and HDPE. This effect is most likely caused by the increased quantity of antioxidants included in the blend, as well as the presence of LLDPE in the mixture. Disintegration was seen after only three months of degradation, and by the 5.5-month point, the mechanical qualities had significantly declined. In general, all films showed a decrease in strength, with PP and the oxo-bio blend sample degrading quickly, followed subsequently by HDPE and LLDPE. The authors concluded that the presence of antioxidant additives did not assist in preventing rapid photo-oxidative degradation, while the presence of a pro-oxidant additive accelerated the samples’ propensity to degrade.

In another study reported by Perez et al. [37], the combined effect of reprocessing and accelerated weathering on acrylonitrile-butadiene-styrene terpolymer (ABS) was investigated. ABS was reprocessed up to 10 times using twin-screw extruder and injection moulding. Accelerated weathering was performed on selected samples by exposing them in a QUV test chamber as per ASTM G-53-84, using alternating cycles of UV (4 h at 59.85 °C) and moisture (100% RH, 4 h at 49.85 °C) for a duration of 600 h. The authors observed that the tensile strength of the recycled samples (10 cycles) exhibited a significant reduction with ageing time. At 50 and 220 h of exposure, the tensile strength of the recycled samples decreased by 48% and 73%, respectively. This was attributed to the photolysis of the methylene bond in the trans-1,4-polybutadiene structure resulting in the formation of allylic radical polymer chains that undergo further reaction to yield hydroperoxides, ketones, and esters. Additionally, crosslinking reactions can also take place in the polybutadiene phase resulting in higher fragility and a lower tensile strength.

The effect of different types of nanoparticles (SiO_2_ nanoparticles, carbon nanotubes, and untreated and treated montmorillonite) on the UV stability of HDPE was investigated by Grigoriadou et al. [38]. Thin film samples were exposed to UV irradiation (chamber BS-09) at 280 nm at a constant temperature (25 °C) and a constant relative humidity (50%) for several days. It was observed that in all the samples, the presence of nanoparticles resulted in a stabilization effect on HDPE, and in samples containing SiO_2_ and MMT, the tensile strength reduction was found to be lower than 50%. The tensile strength and Young’s modulus of the samples increased by up to 100 h of early UV irradiation, which was attributed to enhanced crystallinity in some samples. Long-term UV exposure caused macromolecular chain scission, which degraded the mechanical properties of the materials and caused surface imperfections and holes in the films.

The co-relation between natural and artificial weathering has been attempted by a number of researchers [39]. Philip and Al-Azzawi [40] compared the effect of degradation on recycled polyethylene terephthalate (PET), under both natural and accelerated weathering conditions. The natural weathering conditions comprised the samples exposed to the sun for a period of up to 13,000 h. The artificial weathering conditions included exposing samples to 8 h of UV radiation at 60 °C and 4 h of condensation at 40 °C in a 12 h cycle. The recycled PET samples were assessed at various intervals, including 0, 250, 500, 750, 2000, and 9000 h. The main observations were that after 5000 h of exposure, the impact strength decreased. Colour measurements indicated a colour transition shift beyond 2000 h due to surface roughness changes caused by degradation. The authors co-related subjecting samples to 25 days of accelerated weathering with 1 year under natural weathering. Other researchers [41] performed a comparison study using polypropylene oyster spat collectors, performing in situ weathering for 55 months and exposure to artificial weathering for five months. The authors observed that five months of ageing under the artificial weathering conditions was comparable to 4.4 months of natural sunlight exposure. The effect of photooxidation was also observed to be higher in air than in seawater.

### 4.2. Biocomposites

Biocomposites comprise organic fillers (plant fibres, biomass, waste residues) incorporated in conventional plastics or biopolymers. Numerous studies have investigated the effect of weathering on the properties of biocomposites [42,43,44]. Turku and Karki [42] investigated the effect of fire retardants (aluminium trihydrate, zinc borate, melamine, graphite, titanium dioxide) on the durability of wood fibre-reinforced polypropylene composites. The samples were subjected to weathering tests in a Q-Lab Xenon Test Chamber under the following conditions: 102 min of UV irradiation (with an average irradiance of 0.51 W/m^2^ at 340 nm) at a chamber temperature of 38 °C and (50 ± 10)% relative humidity, followed by 18 min of water spraying. The tensile strength and modulus of wood-plastic composites (WCPs) after weathering were found to decrease due to fibre-matrix interfacial debonding and the removal of hydrophilic materials from the composite. The control sample just consists of pigment, whereas the other samples comprise a variety of fire retardants. The pigment’s UV radiation-blocking capabilities caused minor colour changes even in the reference.

The impact of weathering on tensile properties can be reduced by incorporating synthetic fibres along with plant fibres. In a study on hybrid fibre composites, Abdullah [45] found that polyoxymethylene composites containing hybridizing fibres (kenaf-PET) exhibited a lower decrease (2%) in mechanical properties compared to biocomposites containing single fibres (a decrease of 50%). The study suggests that hybrid fibre biocomposites made from kenaf and PET fibres could be a better option for the manufacturing of automotive components compared to using kenaf fibre biocomposites.

**Figure 2:**
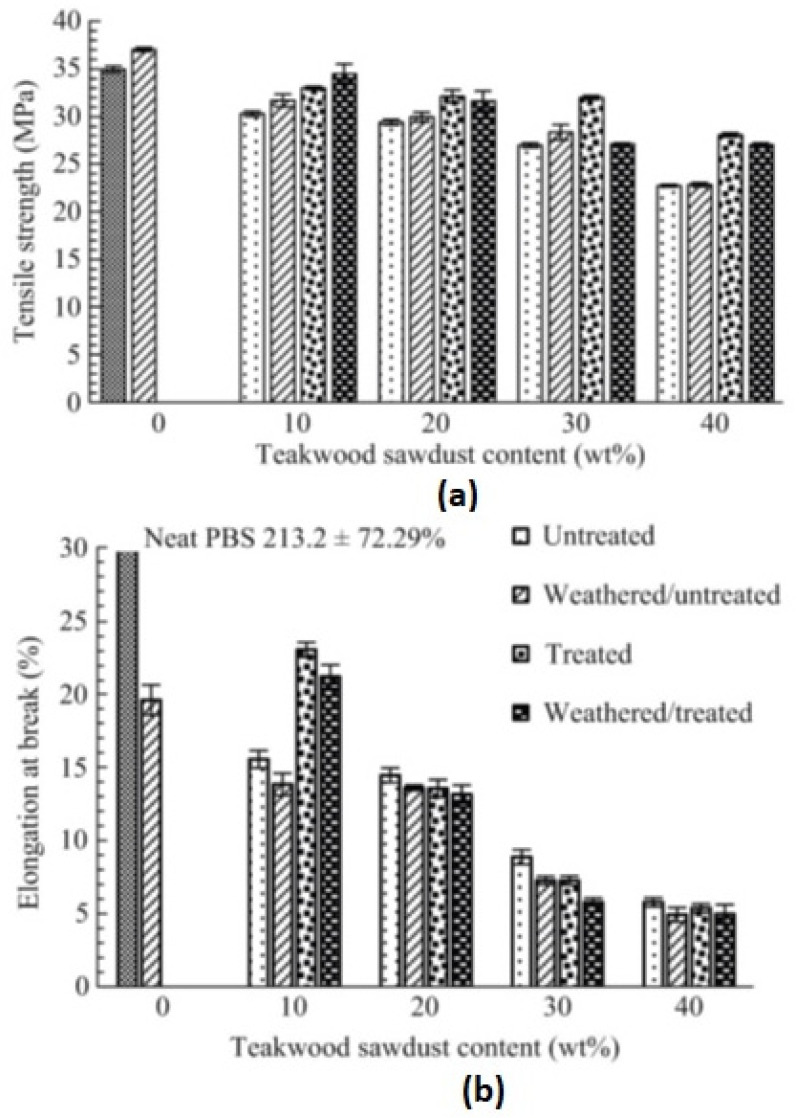
Tensile properties of neat PBS and PBS/teakwood composites: (**a**) tensile strength; (**b**) elongation at break [46].

The durability of teak sawdust-reinforced PBS composites was studied by Hongsriphan et al. [46]. Weathering experiments were carried out as per ASTM-G154 [47] for five cycles. It was observed that after weathering for 60 h, there was a slight increment in Young’s modulus and tensile strength of neat PBS; however, the strain values reduced by 94% (Figure 2). The drastic reduction in flexibility indicated deterioration in the amorphous region of the PBS matrix. In the case of composites, similar results were observed, being attributed to the cyclic expansion and contraction of the samples.

The addition of bio-based fillers such as lignin in plastics and biopolymers is seen to retard the effect of photodegradation. In an interesting study, Pucciariello et al. [48] observed that incorporating straw lignin into LDPE and PS blends reduced the effects of UV degradation. The neat samples and blends were irradiated for 48 h using a 15 W UV lamp. The authors suggested that the degradation process is retarded due to the presence of phenolic groups in lignin, which have antioxidant and radical scavenging properties. Following irradiation, the mix, which contained 20% lignin and 80% polystyrene, exhibited an increase in average molecular weight, which was particularly evident at higher lignin concentrations, indicating a potential protective effect against photo-oxidation. Similar results were observed by Spiridon et al. [49], who utilised two forms of lignin, organosolv and lignoboost, with PLA and exposed the samples to accelerated weathering using a mercury lamp for 600 h. In addition, the authors observed that composites containing 7% of lignin retained much of their initial mechanical properties after accelerated weathering compared to the neat PLA matrix. The results showed that the organosolv lignin composites derived from hardwood displayed the best mechanical properties before and after ageing.

The incorporation of lignin in polybutylene adipate terephthalate (PBAT) films and its effect on improving photostability was studied by Botta et al. [50]. The ageing tests comprised exposure cycle conditions of 8 h of light at 55 °C, followed by 4 h of condensation at 45 °C and an RH of 40 ± 3%.

**Figure 3:**
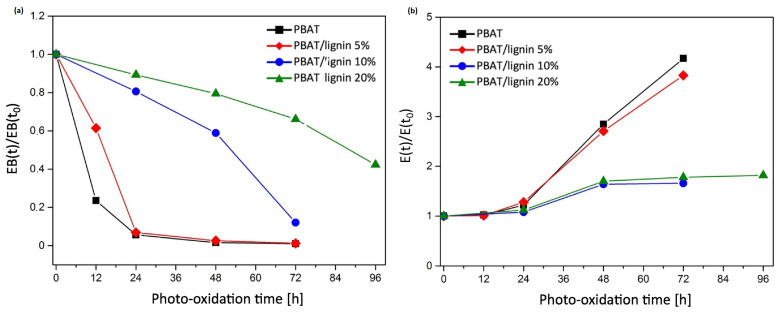
(**a**) Dimensionless elongation at break and (**b**) dimensionless elastic modulus as a function of photo-oxidation time.

In terms of mechanical performance, Figure 3 depicts the variation of dimensionless elongation at break (defined as the ratio of deformation at break at a given irradiation time-point, EB(t), to that of the unaged samples, EB(t_0_)) and elastic modulus with photo-oxidation time. After 24 h, a quick decrease in the dimensionless deformation at break was observed for neat PBAT films. In the case of the lignin-containing composites, the decrease was significantly lower. The degradation duration for PBAT was roughly 10 h, during which its characteristics decline by 50%, and it was shown to extend to 90 h for PBAT containing 20% lignin. The dimensionless elastic modulus of all samples increases with exposure duration, which can be attributed to increased crystallisation due to photo-degradation and the development of crosslinks in the amorphous phase, which stiffens the polymer matrix. PBAT reinforced with lignin at 10 and 20% loading showed a slower elastic modulus increase compared to PBAT containing 5% lignin.

**Figure 4:**
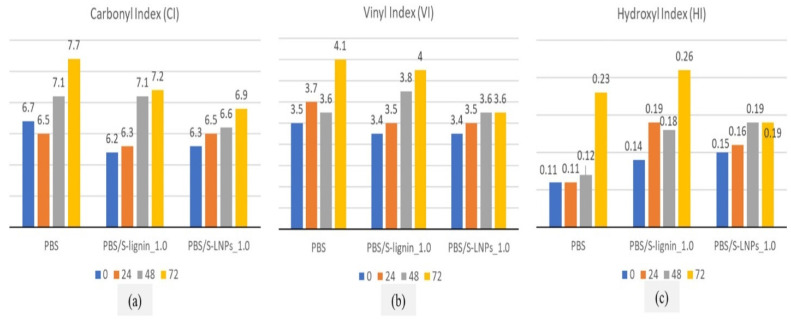
Carbonyl, vinyl, and hydroxyl indices of neat PBS and its composite films at various accelerated weathering times of 24, 48, and 72 h.

The durability of lignin nanoparticle-reinforced PBS films was studied by Hararak et al. [51]. The authors observed that after ageing the films, the carbonyl index (CI), vinyl index (VI), and hydroxyl index (HI) of the samples increased with exposure time, as seen in Figure 4a, Figure 4b and Figure 4c respectively. The increase in CI, VI, and HI was attributed to the formation of carboxylic end groups, ketones, hydroperoxide, and hydroxyl products. In the case of lignin-reinforced PBS composites, the CI, VI, and HI values were lower, as the incorporation of lignin delayed the photo-degradation effects.

**Figure 5:**
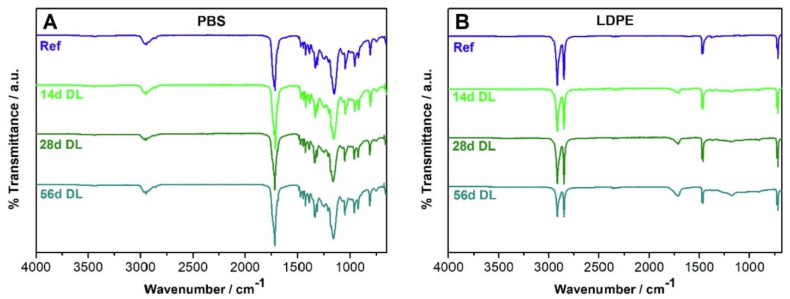
FTIR spectra for PBS (**A**) and LDPE (**B**): untreated reference (Ref) samples and those photo-aged to DL for 14, 28, and 56 day (stacked).

The surface degradation processes of accelerated and natural ageing on PBS and LDPE injection-moulded plates was investigated by Fritz et al. [52]. Ageing experiments entailed 14, 28, and 56 days of daylight (DL) exposure and 7 days of UV irradiation. FTIR spectroscopy was used to monitor the changes in DL aged and unaged samples. For PBS, even after 56 days of exposure, no significant changes could be seen in the FTIR. In the case of LDPE, the study results confirmed the formation of distinctive absorption bands. The aged sample exhibited discrete absorption peaks at 710 and 719 cm^−1^, 2847 and 2915 cm^−1^, 1462 and 1472 cm^−1^ as seen in Figure 5.

Amongst the different biopolymers, a large number of weathering studies have focused on PLA and its biocomposites, as it has the most potential to replace conventional plastics, mainly due to its technical properties [1]. The addition of fibres in PLA is generally seen to reduce tensile properties. This reduction is mainly due to degradation in the amorphous structure, molecular backbone cleavage, weak interfacial adhesion between PLA and fibres leading to debonding, moisture absorption, and swelling resulting in loss of strength. The strength is retained or even increased after ageing, which is due to a nucleating effect of fibres, resulting in higher strength [53]. The incorporation of nanoclays in semi-crystalline PLA results in the formation of intercalated or exfoliated structures, which act as barriers and resist weathering effects [54]. In PLA, the primary degradation mechanism is the hydrolysis of ester bonds, which is accelerated by increasing temperatures. The main degradation products are carboxylic end groups, ketones, hydroperoxides, and hydroxyl products. Additionally, the addition of nano clays results in the formation of an intricate labyrinthine network that slows down the diffusion of water, leading to increased resistance to accelerated weathering [55].

Buffum et al. [56] investigated the effect of UV exposure on the mechanical characteristics of seven types of cutlery made from waste fibres and biopolymers. The samples included wheat straw, fibre pulp, potato starch, bamboo-bulrush-wheat straw mix, bagasse, bagasse-bamboo mix, and PLA. The samples were irradiated with UV light for a period of 15 min at a wavelength of 360 nm. The findings revealed a significant decrease in the mechanical strength of PLA exhibiting a minimum of 50% reduction in tensile strength and tensile modulus. The authors observed that the cellulose-based material (bagasse) was found to be more durable than the starch-based material (wheat straw) and PLA. The effect of natural weathering on PLA biocomposites comprising rice starch (RS) and epoxidized natural rubber (ENR) was studied by Yew et al. [57]. The experiment entailed exposing samples to a mean temperature and a mean relative humidity of 29 °C and 70% for 2 months. The authors observed that the tensile property retention for rice starch composites was higher, while the addition of ENR resulted in severe degradation due to the oxidation process of unsaturated sites within ENR.

**Figure 6:**
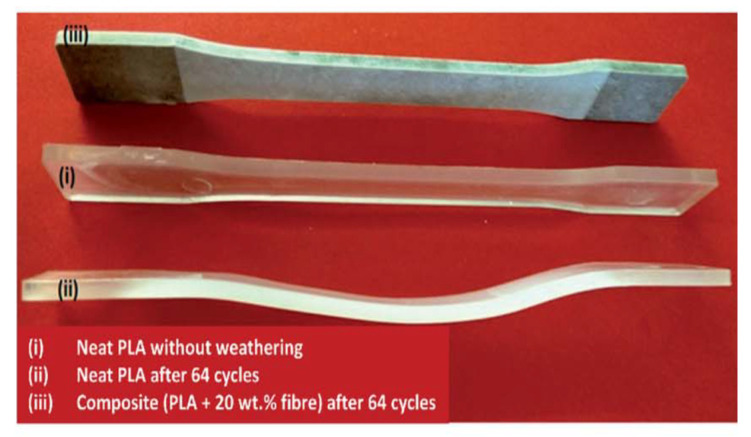
Effect of accelerated weathering ageing cycles on PLA and its biocomposites: dimensional changes [58].

The photodegradation effect is dependent on the type of plant fibres used in composites. In an interesting study by Chollakup et al. [58], the extent of photodegradation of palm fibre-reinforced PP was found to be less than that of pineapple-leaf fibre (PALF)-reinforced PP. This was attributed to the higher lignin content in palm fibres compared to PALF, providing protecting against photodegradation. The performance of hemp fibre-reinforced PLA biocomposites after multiple cycles of UV exposure of accelerated weathering have been investigated by Sawpan et al. [59]. It was found that the mechanical properties of the samples were decreased when increasing the test cycles. For composites containing 30 wt% hemp fibres, the tensile strength decreased by 80% after 64 cycles of weathering (768 h). This was attributed to higher levels of water absorption at high fibre loadings, leading to leaching of materials from the fibre and PLA interface and the formation of cracks. As seen in Figure 6, the neat PLA samples exhibited significant warping after 64 cycles of accelerated weathering, while the PLA composites maintained their dimensional stability due to the presence of hemp fibres.

**Figure 7:**
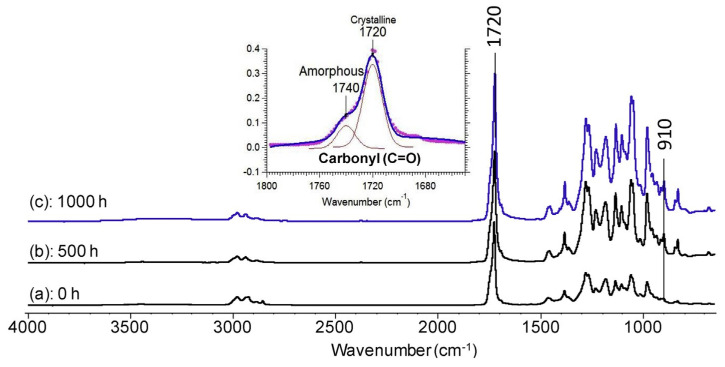
FTIR spectra of PHBV films: un-weathered control (a) and weathered for (b) 500 h and (c) 1000 h.

In the study reported by Wei and McDonald [60], the degradation mechanism of PHBV during accelerated weathering was monitored by FTIR as seen in Figure 7. The samples were exposed to a repeated 2 h cycle of radiation followed by exposure to water for a duration of 1000 h. The authors observed the increased intensity of the ester carbonyl (C=O) and C-O stretching bands after 500 h of weathering, indicating the photo-oxidation of PHBV. These findings showed that the mechanism of PHBV degradation involves Norrish Type II reactions, which resulted in the synthesis of vinyl groups and unsaturated C=C bonds.

**Figure 8:**
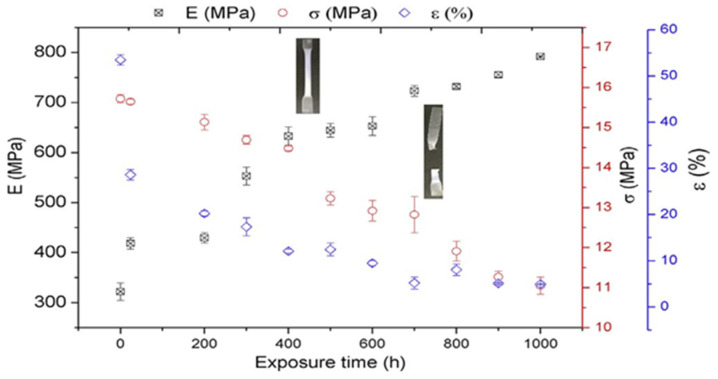
Tensile property (E, ε, and σ) changes of PHBV with exposure time (0–1000 h).

The changes in tensile properties of PHBV are shown in Figure 8. It was observed that the tensile and elongation values decreased with weathering time. However, after 24 h of ageing, the modulus increased by 30%. After 700 h of exposure, the samples were found to completely fragment.

Numerous studies reporting on accelerated weathering experiments on biopolymers reinforced with different types of bio-fillers are listed in Table 1.

### 4.3. Photodegradation in Marine Environment

Plastics can enter the marine environment due to improper disposal or spills during production or transport, creating a major hazard to the marine ecosystem. Plastics in seawater either float or sink and are deposited on the seabed, depending on the density of the material. Lightweight floating trash is commonly swept ashore and buried in tidal sandy sediment [72]. The effect of photodegradation on plastics is reduced in seawater due to oxygen availability, lower temperature, and the fact that the rate of hydrolysis of most polymers is very low in the ocean [73].

The study reported by Wu et al. [74] investigated the photodegradation of polypropylene (PP) in both in seawater and freshwater environments. The PP samples were exposed to ultraviolet (UV) radiation, simulating the natural condition of sunlight exposure in the marine environment. The degradation of PP was investigated by measuring the changes in its chemical and physical properties, including molecular weight, morphology, weight loss, etc. The obtained results indicated that the degradation of PP was significantly greater in seawater compared to freshwater. After 60 days of exposure to UV radiation, the weight loss of PP in seawater was 39%, compared to 21% in freshwater. The study also revealed that the surface morphology of PP samples exposed to seawater was more severely damaged than that of the PP samples exposed to freshwater. The presence of salts and dissolved organic matter was identified as the main factor contributing to the accelerated photodegradation of PP in seawater. In addition, the researchers observed that the degradation of PP in seawater produced more microplastic particles in the freshwater environment.

A similar study reported by Liu et al. [75] investigated the photodegradation of polystyrene (PS) in the marine environment. The PS samples were exposed to UV radiation and seawater separately, as well as in combination, and the degradation of PS over time was monitored. The results revealed that both UV radiation and seawater significantly accelerated the photodegradation of PS, with the combined effect being most pronounced. After 60 days of exposure to UV radiation and seawater, the weight loss of PS was found to be 63%, compared to 15% in the PS samples exposed to UV radiation or seawater alone. The morphology was also observed to be more severely damaged than that of the PS sample exposed to UV radiation or seawater. Furthermore, the study revealed that the photodegradation of PS in the marine environment led to the formation of microplastics and the release of toxic chemicals, including bisphenol A (BPA) and styrene monomer.

Naik et al. [76] conducted investigations to simulate the weathering of four samples namely, HDPE, high-impact polystyrene (HIPS), Nylon 9 and PP. The samples were placed in quartz vials with 5 mL of synthetic water and weathered for 5 days in a Rayonet RPR100 photochemical reactor. The results showed that the simulated weathering generated microfibres from HDPE and Nylon 6, while HIPS and PP did not physically degrade. The same technique was applied to sediment samples collected near large plastic manufacturers, which were found to contain microplastics composed of PP and PE.

## 5. Analysis of VOC Emissions

Several reviews related to the degradability of polymeric materials have been published [9,77,78]. However, there are very few reviews on the characterisation of VOCs. The characterisation of VOCs is crucial because these chemicals have adverse effects on the environment and human health. The chemical characterisation of VOCs typically involves the separation of components present in the gas mixture using a gas chromatography column (GC) and the characterization of each molecule as it reaches the detector of the mass spectrometer (MS) [79].

### 5.1. Ambient Mass Spectrometry Analysis

Ambient mass spectrometry analyses are conducted under normal environmental conditions and involve the desorption and ionization of chemicals directly from the surface. Two common approaches for ambient mass spectrometry are Direct Analysis in Real-Time (DART-MS) and Desorption Electrospray Ionization (DESI-MS). Proton Transfer Reaction (PTR-MS) and Selected Ion Flow Tube Mass Spectrometry (SIFT-MS) are also ambient MS techniques that can be used for the real-time assessment of VOCs during the manufacturing process [80]. For instance, [81] monitored VOCs emitted during the degradation of macro- and microplastics using SIFT-MS. The macro and microplastics were derived from PP, PE, and PET. The results suggested that the degradation of these materials emits alcohols with a chain length of two carbon atoms, carboxylic acids with a chain length of four carbon atoms, aldehydes with a chain length of six carbon atoms, and ketones with chain lengths of eight carbon atoms. This technique is regarded as one of the advanced technologies providing fast (it takes less than 2 min to analyse VOCs) and reliable information on the analysis of VOCs. Even though this technique is promising for the future analysis of VOCs, there are still improvements needed to be made. These include the creation of a database to accommodate many other polymers, which will improve the accuracy of the results. In addition, this technique can be coupled with other analyses, such as thermal analysis and spectroscopy, to provide a broad understanding of the degradation process of polymers.

### 5.2. Gas Chromatography-Mass Spectrometry (GC-MS) Analysis

In the GC-MS analysis, VOCs in a gas mixture are separated and identified based on their interaction with the stationary phase. The retention rate of a molecule depends on its interaction with the stationary phase, with stronger interactions leading to higher retention. An inert gas transports the analytes through the capillary column to the detector, generating a mass spectrum for selected molecules [82].

Two common options for sample pre-treatment are headspace techniques and solvent-based methods [83]. The liquid and gas phases can coexist in closed systems. A liquid sample can be analysed by allowing a volume of vapor to develop above it (known as the sample’s headspace). Once equilibrium is reached, the concentration of the analyte in the mixture’s headspace is determined, using gas chromatography [84]. Headspace techniques may be more widely used for VOC identification due to their environmental friendliness, shorter-sample pre-treatment time, and greater sensitivity compared to solvent-based techniques [79].

### 5.3. Headspace Techniques

There are several solvent-free approaches for VOC extraction from polymers, including static headspace, thermal desorption, headspace solid-phase microextraction (HS-SPME), headspace stir bar sportive extraction (HS-SBSE), and headspace solid-phase dynamic extraction (HS-SPE). HS-SPME is the most widely used technique, applied to the investigation of VOCs in synthetic polymers as well as in physical patterns such as weather-stripping around windows, carpets, plastic flooring, and fibreglass resin. Thermal desorption is also widely used, especially for polyolefins and PET materials. Static headspace is less commonly used due to its lower sensitivity, as VOCs can be mixed with air in the extraction chamber during extraction. HS-SPDE (headspace solid-phase dynamic extraction) is a potential choice for VOC extraction from polymers, as it has a larger adsorbent surface and higher sensitivity compared to HS-SPME. HSSE (headspace sportive extraction) has not been widely used in polymer applications but has shown positive findings in food aroma analysis, such as olive oils, indicating its potential for VOC extraction from polymeric matrices [85,86].

### 5.4. Solvent Extraction Techniques

Solvent extraction methods are commonly used for the analysis of VOCs in polymers, and there are several techniques available. These methods rely on the transfer of VOCs from the polymer matrix to a solvent phase for analysis. Some of the solvent extraction methods used for VOC analysis in polymers include stirring, dissolving, supercritical fluid extraction (SFE), accelerated solvent extraction (ASE), microwave-assisted extraction (MAE), ultrasound-assisted solvent extraction (USE), Soxhlet extraction, solvent-assisted flavour evaporation (SAFE), and steam distillation [79].

SFE, which is comparable to USE, is another solid–liquid extraction method. To maintain the supercritical state, the extraction is carried out with a supercritical fluid, typically carbon dioxide, at a specified temperature and pressure [87]. Supercritical CO_2_ is a non-toxic solvent that can be used to extract non-polar to moderately polar molecules. Using methanol as a polar modifier improves polar compound solubility and increases non-polar chemical extraction ability [88].

ASE, also known as Pressurized Liquid Extraction (PLE), is a solid-liquid extraction process that is extremely efficient. To accelerate extraction kinetics, the solvent is kept under high pressure and the temperature is raised over the solvent’s normal boiling point. This high pressure forces the solvent into the matrix pores, assisting in the extraction process [89].

MAE is a popular approach in which electromagnetic radiation heats the solvent or sample, achieving quick and homogeneous heating [90]. The dielectric constant of the solvent affects efficiency, and MAE eliminates weak hydrogen bonds and enhances solvent penetration into the sample matrix, hence increasing analyte solubility. MAE uses solvent mixes that contain at least one microwave-absorbing unit. MAE heats the sample while keeping the solvent cool when applied to non-polar solvents, which improves temperature-sensitive molecules [91].

The solvent extraction techniques aim to accelerate the extraction process and increase the limit of detection of VOCs by using higher temperatures, pressure, enhanced shaking with sonification, recirculating the solvent to increase the solute concentration in the extract, applying supercritical fluids, or isolating the volatiles. Among the commonly used solvent extraction techniques are SAFE, MAE, polymer dissolution, flask-shake extraction, SFE, Soxhlet, USE, ASE, and water distillation. For example, SAFE has been used to remove odour-active substances from various plastics, such as toys and post-consumer heterogeneous plastics [92].

### 5.5. Thermal Desorption–Gas Chromatography–Mass Spectrometry

Thermal desorption-gas chromatography-mass spectrometry (TD-GC-MS) is a widely used analytical method for analysing the release of volatile organic compounds (VOCs) during weathering of polymers. This method involves heating the polymer sample in a controlled environment to release VOCs, which are then analysed using GC-MS [93]. TD-GC-MS allows for the simultaneous analysis of a wide range of VOCs, including those that are difficult to detect using other techniques. It is highly sensitive, with detection limits in the low parts-per-billion range. TD-GC-MS can provide valuable information on the types and number of VOCs released during different stages of weathering. The emission of VOCs from various polymeric materials, such as polystyrene (PS), polyurethane (PU), cellulose acetate (CA), cellulose nitrate (CN), PE, PP, rubber, poly (vinyl chloride) (PVC), and polycarbonate (PC) were analysed by TD-GC-MS in the study reported by Materazzi and Vecchio [94] (2011). For example, they reported that PS emits styrene and ethylbenzene, whereas PU emits propylene glycol, 1.2 dimethyl hydrazine, propyl hydrazine, dipropylene glycol monomethyl ether, dimethyl phthalate, and diethyl phthalate. The TD-GC-MS method has proven to be effective in separating and identifying volatile organic compounds (VOCs) from various polymers following their degradation.

The advantages and disadvantages of the different techniques are presented in Table 2.

### 5.6. Studies on VOC Emissions in Plastics and Biocomposites

In a recent study, the volatile organic compounds (VOCs) present in coffee ground straws (CGSs), polylactic acid straws (PLASs), and polypropylene straws (PPSs) were characterized using headspace—solid-phase microextraction and migration assays by Li et al. [95]. The authors observed the presence of a number of VOCs in the samples; the VOCs identified in CGS registered the highest levels of genetic toxicity, which were higher than those of the PLAS and PPS samples.

**Figure 9:**
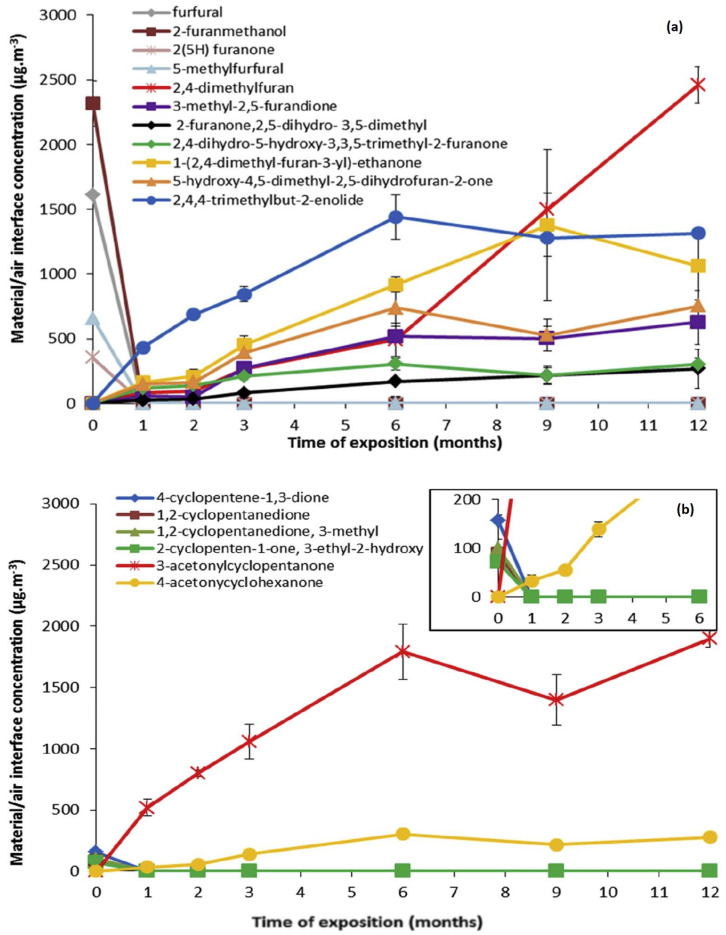
Variations of major furan (**a**) and cyclic ketones (**b**) VOCs released by PP30 over the exposition (PDMS/DVB/CAR SPME fibre sampling at 80 °C).

Badji et al. [96] studied the influence of weathering under windshield glass on VOCs release in hemp fibre-reinforced PP biocomposites in comparison with neat PP. Samples were exposed for a year in the South West of France (Pau) and VOC analysis was performed using gas chromatography coupled to mass spectrometry and flame ionization detections. The authors observed a significant increase in oxygenated compounds for aged biocomposites, as seen in Figure 9. Amongst the chemicals detected from PP30 (PP + 30% hemp fibres + 2% MA-PP) furfural and 2-furanmethanol were seen to be emitted by unaged samples. The furan chemicals originated from the ring-opening of polysaccharides, followed by further cyclization. Amongst the ketonic VOCs, 3-acetonylcyclopentanone was found to be predominant and to mainly originates from hemicelluloses in hemp fibres. In terms of health impacts, the emitted compounds were lower than vehicle indoor air quality (VIAQ) limit values and would comply with VIAQ regulations.

The emissions of VOCs from degraded plastic debris and microplastics have been a topic of current interest [81]. Lomonaco et al. [97] used headspace (HS) sampling followed by needle trap microextraction (NTME) and gas chromatography/mass spectrometry (GC–MS) for profiling of VOCs from conventional plastics, such as LDPE, HDPE, PET, PP, and PS. The authors observed that of all the aged samples, poly (ethylene terephthalate) emitted insignificant quantities of ketones and aromatics while high levels of aromatic substances, lactones and esters were emitted by polyolefins and PS, respectively. The authors also checked the VOCs emitted from plastic debris collected from beaches and observed the emission of lethal substances such as acrolein, benzene, methyl propenyl ketone and methyl vinyl ketone,

The photo-ageing process and emission of VOCs from polystyrene microplastics was investigated by Wu et al. [98]. The authors observed that after 30 days of exposure to UV irradiation, the total amount of VOCs emitted increased by 2.4 times to those samples stored in darkness. Of the VOCs detected, benzene and toluene exhibited increasing levels with irradiation while styrene and 2-propylebenezene concentrations decreased over time.

In a recent study conducted by Wu et al. [99], the authors analysed the volatile organic compounds (VOCs) generated during the UV [A (365 nm) and C (254 nm)] irradiation of polyethylene (PE) and polyethylene terephthalate (PET) for 30 days in water. The findings revealed that UV-A-derived VOCs in PE primarily comprised alkenes and alkanes, while UV-C-derived VOCs included alcohols, aldehydes, ketones, carboxylic acids, and lactones. For PET, both UV-A- and UV-C-derived VOCs included alkenes, alkanes, esters, and phenols. The toxicological analysis showed that dimethyl phthalate from PE and 4-acetylbenzoate from PET were identified as VOCs with the highest potential toxicity. The results also indicated that PE under UV-C irradiation can release toxic VOCs up to levels of 102 µg g^−1^.

In an interesting study, researchers investigated the laboratory-generated fumes from recycled plastic-modified asphalt [100]. The recycled plastics used were r-LDPE, r-ABS, r-PET, and commingled plastics, and VOCs emissions were analysed by GC-MS studies. The authors observed that the use of ABS at 180 °C resulted in higher levels of VOCs and polycyclic aromatic hydrocarbon (PAHs) emissions, indicating a physical degradation of the plastics at high working temperatures. All other samples exhibited reduced VOCs and PAHs at all working temperatures. The authors do emphasise that the observed results are not indicative of an onsite measurement and that several factors, including speed, the direction of the wind and temperature, need to be considered for a comparison analysis against actual onsite measurements.

## 6. Conclusions

The photo-degradation of polymers can result in changes in mechanical, physical, and chemical properties of polymers, subsequently leading to a reduction in its lifespan and performance. Amongst the different types of polymers, the most stable to photodegradation are the polyfluorocarbons, PVDF, PTFE, silicones, and PMMA. These polymers do not absorb UV light and are difficult to oxidize. In the case of thermoplastic polyolefins (polyethylene and polypropylenes) photodegradation can result in surface cracking upon prolonged exposure. Photodegradation in the marine environment is not as drastic as on land due to lower temperatures and availability of oxygen.

Artificial weathering is a very useful technique to evaluate the comparative degradation of the materials in a reasonable timeframe. One of the main challenges in weathering studies is that only limited studies co-relate research to the real-time ageing evolution and effective service life of the materials. In the case of biocomposites, the extent of photodegradation effect is dependent on the type of plant fibres and the interfacial adhesion between the fibres and biopolymer matrices. Amongst the different bio-based fillers, the incorporation of lignin in biopolymers is seen to retard photodegradation due to phenolic groups acting as antioxidants and radical scavengers. Plant fibres are generally seen to lower the tensile properties of composites after ageing due to weak interfacial adhesion leading to debonding, moisture absorption, and swelling, resulting in loss of strength. The use of hybrid fibres (synthetic + plant) is also seen to resist the adverse effects of photodegradation.

During the photodegradation of polymers, there is also the release of volatile organic compounds (VOCs), which have negative impacts on human health and the environment. Polystyrene-based polymers, such as expanded polystyrene foam (EPS) and acrylonitrile butadiene styrene (ABS), for example, are known to emit styrene, a VOC, into the environment. Various types of hydrocarbon gases, including methane, have been reported to be released from environmentally aged low-density polyethylene (LDPE), with the production rate being dependent on the surface-to-volume ratio. Studies on biocomposites show that upon long-term ageing, there are emissions of furans and ketones from the hemicelluloses present in plant fibres. These emissions can vary depending on the composition of the polymer, processing processes, and ambient circumstances.

For specific applications such as biodegradable mulch films, the use of additives can provide protection against UV degradation and retain the mechanical behaviour of the materials; however, there are concerns over leaching of additives into the soil and the resultant toxicity issues. Future research should focus on the development of bio-based and environmentally friendly additives and long-term environmental impact of these products. Another area of research is the co-relation between natural and accelerated weathering through use of mathematical models.

With the implementation of circular economy in various industrial sectors and the increase in recycling rates, as well as the replacement of single-use conventional plastics with biopolymers, it is critical to study the susceptibility of these materials to UV degradation. Material design formulation will need to factor in the environmental impact to ensure long-term performance and sustainability.

## Figures and Tables

**Table 1:** Studies and main results of accelerated weathering on biopolymers containing different types of bio-based fillers and inorganic fillers.

Biopolymer	Bio-Based Filler/Fibre	Accelerating Weathering Conditions	Main Results	References
PLA	Hemp	340 nm fluorescent UV lamp; Cycle: 8 h UV light at 60 °C; 0.25 h water spray without light; 0.75 h condensation at 50 °C. Duration: 768 h	Hemp fibre resulted in lowering of tensile strength of composites by 80% after 64 cycles of weathering (768 h). Neat PLA samples exhibited significant warping after 64 cycles of accelerated weathering while PLA composites maintained their dimensional stability.	[59]
	Alkali treated hemp	340 nm fluorescent UV lamp; Cycle: 1 h UV exposure; 1 min water spray; 2 h condensation all at 50 °C. Duration: 1000 h	After 1000 h, biocomposites containing alkali-treated fibres exhibited lower mechanical properties.	[61]
	Ramie	B313 nm fluorescent UV lamp; Cycle: 8 h of UV exposure at 60 °C; 4 h condensation at 50 °C. Duration: 252 h	Ramie fabric-reinforced PLA composites exhibited improved resistance to ageing.	[62]
	Flax Nonwovens	60 °C temperature and 95% relative humidity (RH) and 30 °C temperature and 95% RH. Duration: 500 h	Tensile properties of composites decrease significantly with temperature.	[63]
	Coir	Cycle: 0.89 W/m^2^/nm, 340 nm, UV-irradiation at 60 °C, and condensation temperature of 50 °C. Duration: 192 h	Coir-PLA composites exhibited significantly higher tensile strength (87%) than PLA after ageing.	[64]
	Bagasse	Temperature cycle of −20 °C to 65 °C (12 h a day at each temperature) for a period of 12 weeks.	After 4 weeks of ageing, mechanical properties increased; tensile strength and modulus increased by 5.25% and 4.31%, respectively. Further exposure resulted in a decrease in mechanical properties.	[65]
	Lignin	Mercury lamp at 30 °C and 60% humidity. Duration: 600 h	Lignin enhanced the weathering resistance when compared to PLA composites.	[49]
	Cellulose whiskers	340 nm fluorescent UV lamp, Cycle: 8 h of UV exposure at 50 °C and 30% RH; 4 h condensation at 40 °C and 100% RH.	Photo-degradation of biocomposites (1440 h) induced hydrolysis, accelerating biodegradation.	[66]
	Talc	310 nm fluorescent UV lamp; Cycle: 8 h UV exposure at 70 °C; 4 h condensation at 50 °C. Duration: 300 h	After 300 h, flexural strength decreased by 80%.	[54]
	Nano clays	Cycle (Rain and Dry): 18 min at 23 °C and 85% R.H; 102 min at 30 °C and 58% RH	After 600 h, the presence of nano-clays increased effects of degradation.	[67]
	Linseed cake	UV light (340 nm, 0.76 W/m^2^, 60 °C) and periodically sprayed with water; 18 min spraying was followed by a 102 min dry period. Duration: 500 h	Incorporation of linseed cake accelerates the degradation of composites.	[68]
	Starch and wood flour	Cycle: 8-h UV exposure; 4 h condensation. Duration: 1200 h	Tensile and flexural strength decreased with ageing.	[69]
PBS-PLA	Coir fibre	Cycle: 0.89 W/m^2^/nm, 340 nm, UV-irradiation at 60 °C, and condensation temperature of 50 °C. Duration: 192 h	Coir fibre-reinforced PLA-PBS samples showed the least reduction in tensile strength after ageing.	[64]
PBS	Teakwood sawdust	60 °C, 8 h irradiation, 4 h condensation per cycle; 5 cycles, 60 h. Total duration: 300 h	Elongation at break of neat PBS was reduced by 94%. In composites, only a slight decrease in E.B was observed.	[46]
	Lignin	8 h of light at 55 °C followed by 4 h condensation at 45 °C and RH 40 ± 3%. Duration: 72 h	In lignin-reinforced PBS composites, the carbonyl, vinyl and hydroxyl index values were lower than those in neat PBS films. Incorporation of lignin delayed degradation effects in PBS.	[51]
PBAT	Lignin	8 h of light at 55 °C followed by 4 h condensation at 45 °C and RH 40 ± 3%. Duration: 72 h	Half time of PBAT films were 10 h and was found to increase to 90 h for PBAT containing 20% lignin.	[50]
	Biochar	Samples were exposed at 70 °C to an irradiance of 0.89 W/m^2^ (at a wavelength λ = 313 nm) and monitored every 24 h.	Presence of biochar delays ageing in PBAT films.	[70]
PHBV	Wood fibre	Natural weathering Duration: 12 months	Real-time sunlight UV exposure resulted in a bleaching effect to all samples. No significant effect on mechanical properties. No mold growth on PHBV samples but samples containing 50 wt% WF showed presence of mold.	[71]

**Table 2:** Advantages and disadvantages of analysis techniques for the evaluation of VOCs.

Technique	Advantages	Disadvantages
Gas chromatography–mass spectrometry analysis	Identification and quantification of a wide range of organic pollutants Highly sensitive High information content: Molecular structural information from the mass spectral fragmentation pattern and elemental compositions of mass signals from high resolution mass spectrometry	Detection limit low for polar, high molecular weight and low volatile products. For Py-GC/MS, only a small amount (a few mg) of samples that can be introduced into the caps (a few mg), the difficulties in performing calibration and quantification, and also the damage to the chromatographic system by the direct introduction of dirty/complex samples.
Ambient mass spectrometry analysis	Measurement of trace levels of VOCs in the gas phase possible No complex sample preparation required, reducing analysis time	Limited applicability for non-volatile or high molecular weight compounds.
Headspace techniques	Allows for the development of green techniques for extraction of volatile compounds in different sample types by eliminating the need for organic solvents Non-selective High extraction speed, stability, simplicity, flexibility, and ease of automation	Low sensitivity. Analytes injected limited to the volume of the syringe. Developing appropriate calibration curves for quantitative measurement of HS components in a solid matrix is challenging.
Solvent extraction techniques	Higher detection limit Suitable for extracting a wide range of VOCs, including polar and non-polar molecules.	Large-scale extractions may require significant amounts of solvent, resulting in higher costs and waste generation. Different solvents may have limited interaction with specific analytes or sample matrices.
Thermal-desorption–gas chromatography–mass spectrometry	Higher detection limit than headspace techniques	Adsorbent beds selectively adsorb VOCs based on their polarity, volatility, and concentration.
